# Effects of Licorice Stem and Leaf Semi-Dry Silage Instead of Alfalfa Hay on In Vitro Rumen Fermentation Characteristics and Dynamic Changes of Rumen Microbial Community in Holstein Cows

**DOI:** 10.3390/vetsci13010108

**Published:** 2026-01-22

**Authors:** Limin Tang, Haonan Liu, Qifeng Gao, Yuliang Sun, Xinyu Xu, Wenghao Li, Dong Lu, Lingfeng Kong, Shudong Liu, Tao Jiang

**Affiliations:** 1 College of Animal Science and Technology, Tarim University, Alar 843300, China; 18196321033@163.com (L.T.); 15199216981@163.com (H.L.); 18809070780@163.com (Y.S.); xuxingyu992024@163.com (X.X.); 13279978915@163.com (W.L.); 17399179368@163.com (D.L.); 13937786941@163.com (L.K.); 2 College of Life Science and Technology, Tarim University, Alar 843300, China; gqfsryd1415926@163.com; 3 Key Laboratory of Livestock and Forage Resources Utilization around Tarim, Ministry of Agriculture and Rural Affairs, Tarim University, Alar 843300, China; 4 Xinjiang Wensu Dairy Cattle Science and Technology Courtyard, Aksu 843100, China

**Keywords:** licorice stems and leaves, semi-dry silage, alfalfa hay, in vitro fermentation, nutrient degradation rate, principal component analysis, microbial composition, dairy cows

## Abstract

This experiment aimed to investigate the effects of replacing alfalfa hay with Glycyrrhiza stem and leaf silage (moisture content: 45%) on rumen in vitro fermentation parameters, nutrient digestibility, and dynamic changes of microbial community composition. In vitro fermentation was conducted with 0% (control group G0A100), 50% (G50A50), and 100% (G100A0) alfalfa hay replaced by semi-dry silage of Glycyrrhiza stems and leaves with 45% moisture content for 72 h. Cumulative gas production (GP), fermentation parameters, microbial community composition at different time points, and post-fermentation nutrient digestibility were determined, with comprehensive evaluation by principal component analysis (PCA) and gray relational analysis (GRA). Results showed that GP of G50A50 and G100A0 was significantly higher than G0A100 at 3 h (*p* < 0.05), and that of G50A50 was significantly higher than the other two groups at 24 h (*p* < 0.05). pH of G50A50 was significantly lower than the other two groups at 3 h (*p* < 0.05). In vitro dry matter digestibility (IVDMD) at 24 h and 72 h, in vitro neutral detergent fiber digestibility (IVNDFD) at 12 h, and in vitro acid detergent fiber digestibility (IVADFD) at 12, 24 and 72 h of G0A100 and G50A50 were significantly higher than G100A0 (*p* < 0.05). PCA comprehensive scores ranked as G0A100 (0.170) > G50A50 (0.141) > G100A0 (−0.311). GRA comprehensive scores ranked as G50A50 (0.792) > G0A100 (0.756) > G100A0 (0.681). LEfSe analysis indicated distinct microbial biomarkers at 72 h, and KEGG functional profiles were highly consistent among groups. Under the experimental conditions, 50% Glycyrrhiza stem and leaf silage is recommended to replace alfalfa hay in dairy cow diets.

## Simple Summary

High-quality forage is in short supply for dairy farming, and alfalfa hay—an important feed—relies heavily on imports, increasing costs for farmers. This study aimed to test whether semi-dry feed made from licorice stems and leaves (a by-product of medicinal licorice production) can replace alfalfa hay for Holstein cows. We conducted in vitro simulations of cow stomach digestion and found that replacing 50% of alfalfa hay with this licorice feed worked best: it promoted feed digestion, maintained stable microbial activity in the cow’s stomach, and kept fermentation performance similar to using pure alfalfa hay. This solution is low-cost and easy to obtain, helping farmers reduce feeding expenses while expanding the types of available forage. It provides practical support for sustainable dairy farming, especially for small-scale farmers.

## 1. Introduction

Forage serves as the foundation for the development of animal husbandry and is influenced by numerous factors such as seasonality, the imbalance between grass and livestock, and production efficiency, which restrict the advancement of the livestock industry [1,2]. The use of plant-based feed alternatives and their bioactive components has garnered increasing attention in the field of ruminant nutrition [3]. The demand for alfalfa in China’s dairy industry is significant, and in recent years, the volume of alfalfa imports has increased substantially. The import prices have varied greatly, leading to increased cost pressures [4,5,6]. The scarcity of high-quality forage opportunities has resulted in decreased livestock product yields, diminished animal health and welfare levels, and economic losses for farmers [7]. Therefore, there is an urgent need for inexpensive and high-quality alternatives.

The global annual production of licorice exceeds 120,000 tons, primarily distributed in regions such as China, Iran, and Central Asia, with Xinjiang being one of its major production centers [8]. In this region, the southern part accounts for over 70% of the licorice resource reserves [9]. The stems and leaves of licorice are agricultural by-products, while the underground roots and rhizomes are used for medicinal purposes. The above-ground stems and leaves, after drying, are utilized as feed. The demand for high-quality roughage in China’s dairy industry continues to rise, with the import volume of alfalfa hay reaching 2.0717 million tons in 2022, at an average import price of 511 USD per ton [10]. The high dependency on imports has led to elevated feeding costs, which particularly burdens the economic situation of small-scale farmers in southern Xinjiang. Notably, licorice stems and leaves have a remarkable cost advantage over alfalfa hay in the southern Xinjiang dairy industry. The market price of locally sourced alfalfa hay in southern Xinjiang is approximately 1467–1760 yuan per ton (including transportation costs within the region), calculated based on the actual transaction price of 4400 yuan for 2.5–3 tons. In contrast, licorice stems and leaves—by-products after harvesting medicinal licorice roots—have a total cost (including on-site collection, semi-dry silage processing, and regional transportation) of only 327–409 yuan per ton, derived from the transaction standard of 6540 yuan for 16–20 tons of finished silage. This significant cost gap (nearly 4–5 times lower than alfalfa hay) makes licorice stem and leaf semi-dry silage a highly promising alternative, effectively alleviating the cost burden of high-quality forage for local farmers in southern Xinjiang. Licorice stems and leaves possess advantages such as low cost, easy availability, high nutritional value, and the ability to stimulate animal feeding. They can serve as a quality roughage to partially replace other high-quality forages or concentrates in livestock feeding, thereby enhancing farming efficiency [9,11,12,13,14]. Research on licorice primarily focuses on the feeding, nutritional value of mixed silage, and feeding effects of its extracts, while there are few reports regarding semi-dry silage [8,15,16,17,18,19,20]. Currently, there is a gap in the optimization of the silage process for licorice stems and leaves, as well as corresponding in vivo and in vitro experiments. Our group previously found that adding silage additives when the moisture content is at 45% yields better results for semi-dry silage of licorice stems and leaves [21].

Therefore, the purpose of this study was to determine the effect of increasing the replacement of alfalfa hay by licorice stem and leaf semi-dry silage in the diet. Specifically, we conducted a comprehensive assessment of the characteristics of fermentation gas production and the rate of nutrient degradation based on principal component analysis and grey relational analysis, and explored the dynamic changes in microbial communities. We hypothesized that partially increasing the substitution of licorice stem and leaf semi-dry silage for alfalfa hay in the diet of Holstein cows produces similar rumen fermentation characteristics due to similar nutrients. We also hypothesized that the rumen microbial flora of Holstein cows is similar and can partially replace alfalfa hay. This study provides data support for subsequent feeding trials, facilitating the promotion of this feed for practical applications. It serves as a reference for improving the shortage of forage in Southern Xinjiang.

## 2. Materials and Methods

### 2.1. Experimental Materials

The semi-dry silage of licorice stems and leaves has been prepared in previous experiments, and it has been determined that the optimal quality is achieved at a moisture content of 45% (The licorice stems and leaves were harvested during the flowering period from the Xin Nong Licorice Pharmaceutical Co., Ltd., in Aral City, China. The stems and leaves were crushed to a length of 2–3 cm, and the silage additive was provided by the Beijing Precision Animal Nutrition Research Center Co., Ltd., in Beijing City, China. An amount of 0.025–0.030 g of the silage additive was added per kilogram of silage feed. The raw materials were packed into 2 L plastic jars, compacted to fill completely, with each jar weighing approximately 1.4 kg. The jar openings were sealed with plastic wrap and tape, and stored in a dark place at room temperature for 60 days) [21]. The alfalfa hay was provided by the Animal Science and Technology Experimental Station of Tarim University in Aral, Xinjiang. Prior to the experiment, both samples were placed in an oven at 65 °C until they reached a constant weight, then ground and passed through a 40-mesh sieve, and stored in a dry and sealed environment. The dry matter (DM) content of the two samples was determined using the method described in GB/T 6435-2014 [22]; the crude protein (CP) content was measured using the Kjeldahl nitrogen method as per GB/T 6432-2018 [23]; the neutral detergent fiber (NDF) content was determined according to GB/T 20806-2022 [24]; the acid detergent fiber (ADF) content was measured using the method outlined in NY/T 1459-2022 [25]; the calcium content was determined following GB/T 6436-2018 [26]; and the total phosphorus content was measured according to GB/T 6437-2018 [27]. Table 1 shows the nutritional levels of the semi-dry silage of licorice stems and leaves compared to alfalfa hay.

### 2.2. Experimental Design

The experiment employed a single-factor experimental design, divided into three groups, Group nomenclature: G0A100 = 0% Glycyrrhiza silage + 100% alfalfa hay; G50A50 = 50% Glycyrrhiza silage + 50% alfalfa hay; G100A0 = 100% Glycyrrhiza silage + 0% alfalfa hay. Each group had five replicates at each time point, along with five blank groups for calibration. This experiment was conducted in accordance with the “Standards for Dairy Cattle Feeding” (NY/T 34-2004) [28], with the diet formulated based on a body weight of 600 kg, a milk yield of 30 kg, and a fat content of 4.1%, maintaining a concentrate-to-forage ratio of 5:5. The composition and nutritional levels of the diet are shown in Table 2.

### 2.3. Experimental Animals

This experiment was approved by the Tarim University Science and Technology Ethics Committee (Approval No: PB20250828001). Three healthy adult Holstein cows were selected for the study. Prior to morning feeding, a gastric tube rumen fluid collector (ANSCITECH, Beijing Jiuyan Technology Co., Ltd., in Beijing City, China) was used to extract rumen fluid from the donor cows orally. The fluid was then filtered through four layers of gauze into a thermos for use in the laboratory.

### 2.4. Measurement of Gas Production and Gas Production Parameter

Accurately weigh 0.2 g of the feed sample to be tested and place it into a 100 mL graduated syringe. Apply a sufficient amount of petroleum jelly evenly inside the syringe to ensure gas tightness and prevent leakage. Prepare artificial rumen fluid using the method described by Menke et al. [29], and mix the rumen fluid with the artificial rumen fluid in a volume ratio of 1:2, while continuously introducing anaerobic CO_2_ until the solution becomes colorless, maintaining the temperature at 39 °C during this process. Add approximately 30 mL of the mixed culture medium into the syringe, expel the air from the syringe tube, seal it, and record the corresponding initial scale value. The culture syringe is then quickly placed into a preheated (39 °C) water bath shaker (SHZ-B, Jiangsu Jinyi Instrument Technology Co., Ltd., in Jiangsu City, China) for incubation. The gas production from each syringe is recorded at 0, 3, 6, 9, 12, 24, 36, 48, and 72 h, and samples are taken accordingly. The gas production is calculated using the formula GP = a + b (1 − e^−ct^), where t represents the fermentation time (h); GP denotes the gas production at time t (mL); a corresponds to the gas production from the rapidly degradable portion (mL); b refers to the gas production from the slowly degradable portion (mL); c is the gas production rate constant (%/h); and a + b indicates the potential gas production (mL). Samples are taken at 0, 3, 12, 24, and 72 h using a syringe and immediately placed in ice water to halt fermentation. Preliminary trials indicate that the changes in indicators at these time points exhibit similar trends to those at adjacent time points. Adding these time points would not provide new information. To reduce the workload and cost of sample processing while ensuring the data quality at critical time points, the settings at 0, 3, 12, 24, and 72 h can adequately reflect the key stages of the entire fermentation process. The fermentation liquid is then analyzed for pH using a pH meter (FE28-standard, Mettler Toledo, in Shanghai City, China), and three of the collected samples are stored at −20 °C for the determination of ammonia nitrogen (NH_3_-N) using the method reported by Searle et al. [30], volatile fatty acids (VFA) using the method by Yang et al. [31], and microbial crude protein (MCP) using the Bradford [32] method with Coomassie Brilliant Blue. Another sample is stored at −80 °C for 16S rDNA gene sequencing analysis of rumen bacteria.

### 2.5. Determination of Nutrient Degradation Rate

The experiment was conducted using a DaisyII incubator (ANKOM, Shenyang Mulun Technology Co., Ltd., in Shenyang City, China), with Holstein cows serving as the rumen fluid donors for in vitro cultivation. F57 filter bags (with a 25 μm pore size three-dimensional structure) were washed with acetone for 3–5 min and then completely dried to remove surfactants, preventing the degradation of microorganisms. Each digestion jar contained 1600 mL of mixed buffer solution and 400 mL of rumen fluid. The mixed buffer solution consisted of Solution A and Solution B in a 1:5 ratio, where Solution A contained 10 g/L of potassium dihydrogen phosphate, 0.5 g/L of magnesium sulfate, 0.5 g/L of sodium chloride, 0.1 g/L of calcium chloride, and 0.5 g/L of urea, while Solution B contained 15 g/L of sodium carbonate and 1 g/L of sodium sulfide. Prior to the experiment, the stirrer was preheated with water at 39 °C. After adding the liquid and samples to each digestion jar, CO_2_ gas was introduced for 30 s, and then the lids were tightly sealed. Samples were taken at 3, 12, 24, 48, and 72 h during the incubation. After the incubation period, the nylon bags were rinsed with water at 39 °C for 5 min and then placed in an oven at 65 °C until constant weight was achieved. The dry matter (DM) content in the feed and residues was determined using the national standard GB/T 6435-2014 [22]; the neutral detergent fiber (NDF) content was determined according to the national standard GB/T 20806-2022 [24]; and the acid detergent fiber (ADF) content was determined using the industry standard NY/T 1459-2022 [25]. Degradation rate (%) = 100 × (a − b)/a. a is the content of nutrients (g); b is the content of nutrients in residue after fermentation for 72 h (g).

### 2.6. Determination of Rumen Microbial Diversity

Genomic DNA from samples was extracted using the TGuide S96 Magnetic Soil/Stool DNA Kit (Tiangen Biotech (Beijing) Co., Ltd., in Beijing City, China) according to the manufacturer’s instructions. The quality and quantity of the extracted DNA were assessed using 1.8% agarose gel electrophoresis, and the concentration and purity of the DNA were determined using a NanoDrop 2000 UV-Vis spectrophotometer (Thermo Scientific, Wilmington, NC, USA). The full-length 16S rRNA gene was amplified using the primers 27F: AGRGTTTGATYNTGGGCTCAG and 1492R: TASGGHTACCTTGTTASGACTT. The PCR products were purified using VAHTSTM DNA Clean Beads (Vazyme, Nanjing, China) and quantified using the Qubit dsDNA HS Assay Kit and Qubit 3.0 Fluorometer (Invitrogen, Carlsbad, CA, USA; Thermo Fisher Scientific, Waltham, MA, USA). Sequencing was per-formed on the PacBio Sequel II platform (Beijing Biomarker Technologies Co., Ltd., Beijing, China) using the Sequel II Kit 2.0.

### 2.7. Comprehensive Analysis Based on Principal Component Analysis

The PCA mathematical model is employed to analyze multiple indicators, with calculations referring to the methods of Liu et al. [33].

F_1_ = P_11_ZE_1_ + P_21_ZE_2_ + ⋯ + P_n1_ZE_m_

F_2_ = P_12_ZE_1_ + P_22_ZE_2_ + ⋯ + P_n2_ZE_m_

F_3_ = P_13_ZE_1_ + P_23_ZE_2_ + ⋯ + P_n3_ZE_m_

F_4_ = P_14_ZE_1_ + P_24_ZE_2_ + ⋯ + P_n4_ZE_m_

In these equations, P_1i_, P_2i_, …, P_ni_ (i = 1, n) are the eigenvectors of the covariance ma-trix Σ of E, and ZE_1_, ZE_2_, …, ZE_m_ are the standardized values of the original variables.

### 2.8. Data Statistics and Analysis

The experimental data were analyzed using SPSS 24.0 software for one-way ANOVA, and multiple comparisons were conducted using the Duncan method. The results are presented as means and standard error of the mean (SEM) (n = 5), with *p* < 0.05 indicating significant differences and *p* ≥ 0.05 indicating non-significant differences. The calculation of degradation parameters a, b, and c was completed through nonlinear regression analysis using SPSS software. Graphing and correlation analysis, as well as principal component analysis, were conducted using SPSS (v24.0) and Origin (v2021). Additionally, data normalization was required prior to principal component analysis. Using the SPSSAU software (v26.0) for gray relational analysis, it is necessary to perform dimensionless processing on the data beforehand. Microbial sequencing data analysis was based on the Naive Bayes classifier in QIIME2 [34] (v2020.6), with OTUs classified and annotated using the SILVA database [35] at a confidence level of 70%. Alpha diversity was calculated and displayed using QIIME2 (v2020.6) and R software (v3.1.1). Beta diversity was assessed using QIIME to evaluate the similarity of microbial communities across different samples. Linear discriminant analysis (LDA) effect size (LEfSe) was employed to test for significant taxonomic differences between groups, with a logarithmic LDA score threshold set at 4.0 for discriminative features. Functional predictions were made using PICRUSt2 (v2.6.2) [36], which aligns feature sequences (16S rRNA) with reference sequences from the Integrated Microbial Genomes (IMG) database to construct phylogenetic trees. This approach integrates KEGG pathway information of genes to predict the overall pathway profiles of the community.

## 3. Results

### 3.1. The Effect of Semi-Dry Ensiling of Licorice Stems and Leaves as a Substitute for Alfalfa Hay on In Vitro Fermentation Gas Production and Gas Parameters

As shown in Table 3, the gas production gradually increased with the extension of fermentation time. After 3 h, the gas production in the G50A50 and G100A0 groups was significantly greater than that in the G0A100 group (*p* < 0.05). After 24 h, the gas production in the G50A50 group was significantly higher than that of the other two groups (*p* < 0.05).

### 3.2. The Effect of Semi-Dry Silage of Licorice Stems and Leaves as a Substitute for Alfalfa Hay on In Vitro Fermentation Parameters

As shown in Table 4, the pH value gradually decreases with the extension of fermentation time. The pH values of the G50A50 group at 3 h are significantly lower than those of the other two groups, while the pH value at 12 h is significantly higher than that of the other two groups (*p* < 0.05). The pH values of the G50A50 group at 72 h are significantly lower than G100A0 group (*p* < 0.05). The NH_3_-N content gradually increases with the extension of fermentation time, with the NH_3_-N content of the G50A50 group at 0 h being significantly lower than that of the other two groups (*p* < 0.05). The MCP content first increases and then decreases with the extension of fermentation time, reaching its maximum at 24 h. The MCP content of the G100A0 group at 0 h is significantly lower than that of the other two groups (*p* < 0.05), and the MCP content of the G0A100 group at 12 h is significantly lower than that of the other two groups (*p* < 0.05).

As shown in Table 5, the content of volatile fatty acids gradually increases with the extension of fermentation time. At 0 h, the acetic acid (AA) content in the G50A50 group is significantly higher than that in the other two groups (*p* < 0.05). At 12 h, the acetic acid content in the G100A0 group is significantly higher than that in the other two groups (*p* < 0.05). Additionally, at 0 h, the propionic acid (PA), butyric acid (BA), and total volatile fatty acids (TVFA) content in the G100A0 group are significantly lower than those in the other two groups (*p* < 0.05).

### 3.3. The Effect of Semi-Dry Silage of Licorice Stems and Leaves as a Substitute for Alfalfa Hay on the Degradation Rate of Nutrients in In Vitro Fermentation

Table 6 shows that the in vitro dry matter degradation rate (IVDMD) of the G0A100 group is significantly higher than that of the other two groups at both 3 h and 12 h (*p* < 0.05). The IVDMD of the G0A100 and G50A50 groups is significantly higher than that of the G100A0 group at both 24 h and 72 h (*p* < 0.05). The in vitro neutral detergent fiber degradation rate (IVNDFD) of the G0A100 and G50A50 groups is significantly higher than that of the G100A0 group at 12 h (*p* < 0.05). Additionally, the in vitro acid detergent fiber degradation rate (IVADFD) of the G0A100 and G50A50 groups is significantly higher than that of the G100A0 group at 12 h, 24 h, and 72 h (*p* < 0.05).

### 3.4. Comprehensive Analysis Based on Principal Components

#### 3.4.1. Correlation Analysis

**Figure 1:**
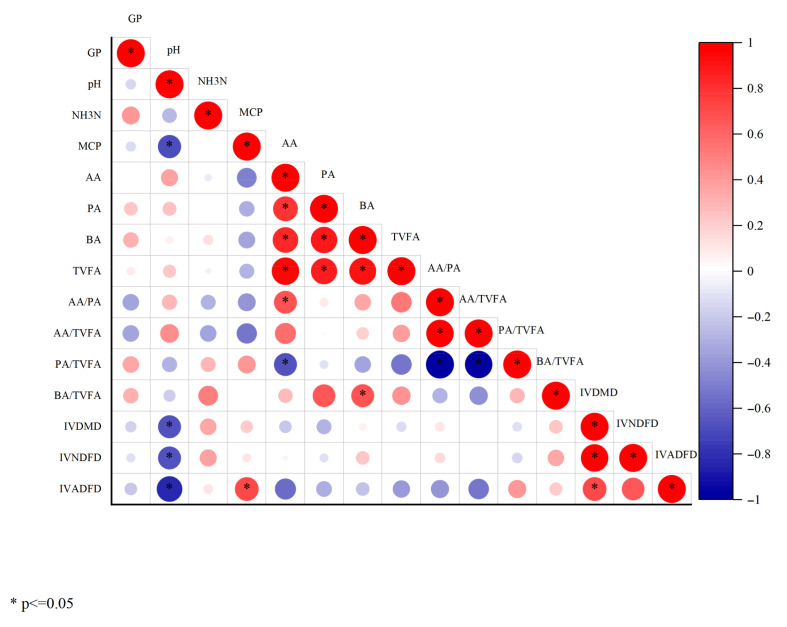
Correlation heatmap of fermentation indicators (*p* < 0.05). Red indicates a positive correlation, and blue indicates a negative correlation, correlation coefficient range: −1 to 1, the deeper the color, the stronger the correlation, The larger the circle, the stronger the correlation. Correlation coefficients were calculated using Pearson correlation analysis, and Benjamini Hochberg method was used for multiple comparison correction. The color scale represents the intensity of correlation.

As shown in Figure 1, TVFA is significantly positively correlated with AA, PA, and BA, which are the main components of TVFA, and their concentrations increase synchronously with TVFA. IVDMD, IVNDFD, and IVADFD are significantly positively correlated with each other, as the dry matter digestibility is primarily determined by fiber digestibility, leading to a high positive correlation among the three. MCP is negatively correlated with VFA, which may reflect that while there is an accumulation of TVFA in the later stages of fermentation, microbial proliferation has entered a stable phase.

#### 3.4.2. Principal Component Analysis and Comprehensive Evaluation

Table 7 shows that the eigenvalues greater than 1 serve as the extraction criteria. Here, the eigenvalues of the first four principal components are 5.781, 3.823, 2.924, and 1.038, all of which exceed 1. The cumulative variance contribution rate reaches 90.437%, indicating that these four principal components can explain over 90% of the information from the original 15 indicators, sufficiently representing the core characteristics of all fermentation indicators. Table 8 indicates that the absolute values of the loadings for GP, pH, MCP, VFA, and IVADFD are greater than 0.6, suggesting that these indicators contribute more significantly to the principal components.

The standardized E_1_, E_2_, …, E_15_ are denoted as ZE_1_ to ZE_15_. Therefore, the principal components are represented as:

F_1_ = 0.082ZE_1_ + 0.262ZE_2_ − 0.238ZE_3_ − 0.284ZE_4_ + 0.355ZE_5_ + 0.177ZE_6_ + 0.177ZE_7_ + 0.317ZE_8_ + 0.307ZE_9_ + 0.301ZE_10_ − 0.318ZE_11_ − 0.088ZE_12_ − 0.230ZE_13_ − 0.193ZE_14_ − 0.338ZE_15_

F_2_ = 0.044ZE_1_ − 0.191ZE_2_ + 0.168ZE_3_ − 0.092ZE_4_ + 0.242ZE_5_ + 0.400ZE_6_ + 0.457ZE_7_ + 0.319ZE_8_ − 0.082ZE_9_ − 0.151ZE_10_ + 0.024ZE_11_ + 0.468ZE_12_ + 0.244ZE_13_ + 0.247ZE_14_ + 0.162ZE_15_

F_3_ = −0.326E_1_ − 0.242E_2_ − 0.060E_3_ + 0.012E_4_ + 0.089E_5_ − 0.216E_6_ − 0.067E_7_ + 0.004E_8_ + 0.368E_9_ + 0.353E_10_ − 0.360E_11_ − 0.140E_12_ + 0.389E_13_ + 0.384E_14_ + 0.255E_15_

F_4_ = −0.644ZE_1_ − 0.505ZE_2_ − 0.057ZE_3_ + 0.442ZE_4_ + 0.084ZE_5_ + 0.027ZE_6_ + 0.003ZE_7_ + 0.068ZE_8_ + 0.110ZE_9_ + 0.109ZE_10_ − 0.079ZE_11_ − 0.146ZE_12_ + 0.123ZE_13_ − 0.225ZE_14_ − 0.047ZE_15_

In the formula, the coefficients represent the feature vectors of the indicators, where F_1_, F_2_, F_3_, and F_4_ denote the scores of the principal components. The variance contribution rates of the initial eigenvalues are used as the weight coefficients for the first four principal components. Thus, the evaluation model for the semi-dry silage indicators of licorice stems and leaves is established, resulting in a comprehensive score calculated as follows:

F = (0.38539F_1_ + 0.25485F_2_ + 0.19495F_3_ + 0.06919F_4_)/0.90437.

As shown in Table 9, the comprehensive scoring of the three ratios of semi-dry ensiled licorice stems and leaves replacing alfalfa hay for in vitro fermentation and degradation indicators is ranked as follows: G0A100 > G50A50 > G100A0.

Based on the previous details of the fermentation indicators, the G0A100 group exhibits the optimal overall fermentation performance among the original alfalfa hay groups. The G50A50 group, which represents the replacement with licorice silage, shows performance closely approaching that of the original group, with 50% being a more feasible substitution ratio. Conversely, the G100A0 replacement group demonstrates the poorest overall fermentation performance. Although the comprehensive score of the G50A50 group is slightly lower than that of the G0A100 group, it demonstrates superior performance in key indicators such as gas production activity and MCP synthesis. This indicates that the 50% licorice silage substitution is a feasible practical solution.

#### 3.4.3. Comprehensive Evaluation of Grey Correlation Degree

As shown in Table 10, the correlation of comprehensive performance among the groups is ranked from high to low as follows: G50A50 (0.792) > G0A100 (0.756) > G100A0 (0.681). Among these, the G50A50 group exhibits the highest correlation, securing the overall first rank.

### 3.5. Effects of Licorice Stem and Leaf Semi-Dry Silage Instead of Alfalfa Hay on Microbial Community in Rumen Fermentation

#### 3.5.1. OTU Analysis

**Figure 2:**
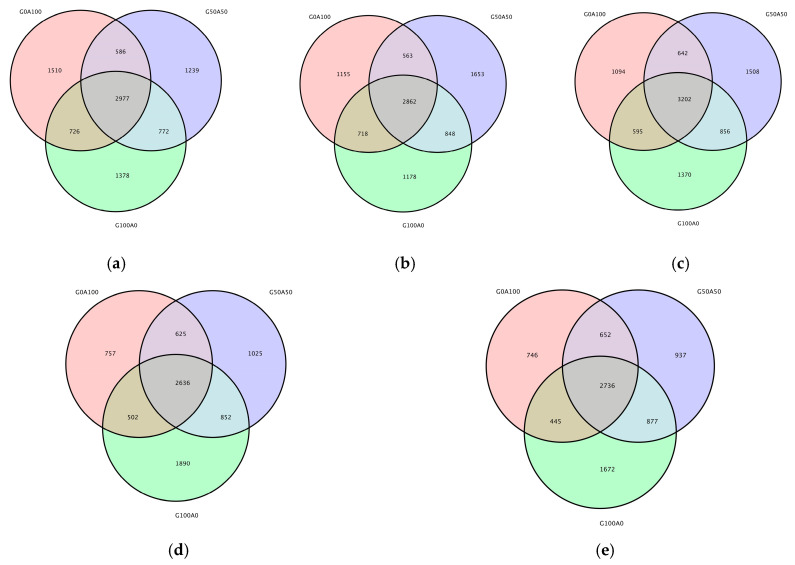
Venn diagram of different fermentation time in each group (in the figure (**a**) 0 h (**b**) 3 h (**c**) 12 h (**d**) 24 h (**e**) 72 h).

As shown in Figure 2, the total number of Operational Taxonomic Units (OTUs) reached its peak at 12 h (3202), indicating a convergence in community structure among different substitution groups during the mid-fermentation phase. The distribution of shared and unique OTUs exhibited a dynamic pattern throughout the fermentation process: a higher number of unique OTUs in the initial stage of fermentation, an increased presence of shared OTUs in the mid-fermentation phase, and a rise in shared OTUs during the later stages of fermentation. This dynamic closely aligns with the rhythm of substrate degradation and microbial adaptation.

#### 3.5.2. Alpha Diversity Analysis

As shown in Table 11, the Shannon index for the G100A0 group at 72 h is significantly higher than that of the other two groups (*p* < 0.05), indicating a significant increase in microbial species diversity in the G100A0 group during the later stages of fermentation. Overall, the substitution of alfalfa hay with licorice silage has a minimal impact on the overall structure of the rumen microbial community.

#### 3.5.3. Distribution of Rumen Microbial Phylum and Genus

Table 12 shows that the dominant microbial communities in the rumen fermentation fluid of each group at the phylum level are Firmicutes and Bacteroidota, with relative abundances ranging from 87.86% to 92.50%, 88.86% to 93.09%, 88.67% to 94.64%, 92.43% to 92.65%, and 88.94% to 93.10%, respectively. At 3 h and 72 h, the Bacteroidota in the G50A50 and G100A0 groups were significantly higher than that in the G0A100 group (*p* < 0.05). At 72 h, the Proteobacteria in the G50A50 group were significantly higher than that in the G100A0 group (*p* < 0.05). At 12 h, the Cyanobacteria in the G50A50 group were significantly higher than in the other groups (*p* < 0.05). At 24 and 72 h, the Desulfobacterota in the G100A0 group were significantly higher than in the other groups (*p* < 0.05). At 3 h, the Spirochaetota in the G50A50 group were significantly higher than in the other two groups (*p* < 0.05).

Table 13 shows that the *Rikenellaceae_RC9_gut_group* in the G0A100 group at 3 h is significantly higher than that in the G50A50 group (*p* < 0.05). At 24 h, the *Rikenellaceae_RC9_gut_group* and *Lachnospiraceae_NK3A20_group* in the G100A0 group are significantly higher than that in the G0A100 group (*p* < 0.05). The *Ruminococcus* in the G0A100 group at 3 h is significantly higher than that in the other two groups (*p* < 0.05). At 24 h, the *Ruminococcus* in the G50A50 group is significantly higher than that in the G0A100 group (*p* < 0.05). The *Lachnospiraceae_NK3A20_group* in the 72 h G100A0 group was significantly higher than that in the other groups (*p* < 0.05).

#### 3.5.4. Beta Diversity Analysis

**Figure 3:**
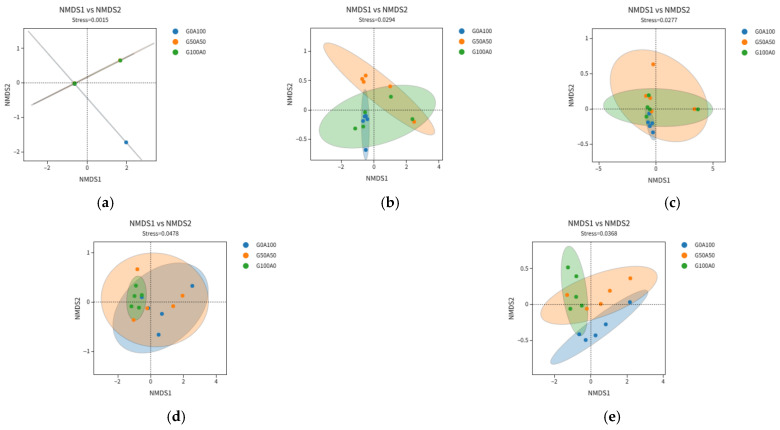
NMDS diagram of each group at different fermentation time (in the figure (**a**) 0 h (**b**) 3 h (**c**) 12 h (**d**) 24 h (**e**) 72 h).

As shown in Figure 3, the Stress values in the NMDS analysis are all below 0.2, indicating that the NMDS analysis is relatively reliable. At 0 h, the three groups of samples are distinctly separated, especially between the G0A100 group and the G50A50, G100A0 groups. At 3 h, there is some overlap among the groups, and the community differences gradually decrease. At 12 h, the three groups of samples show a high degree of overlap, and the community structure tends to become consistent. At 24 h, there is a trend of aggregated distribution in the community, with samples from the G100A0 group clustering together. At 72 h, the differences in community structure among different substitution ratios are again significant. The microbial community structure exhibits dynamic changes characterized by large initial differences, convergence during the mid-fermentation phase, and re-differentiation in the late fermentation phase.

#### 3.5.5. Analysis of Microbial Community Difference Significance, Correlation and Function Prediction

**Figure 4:**
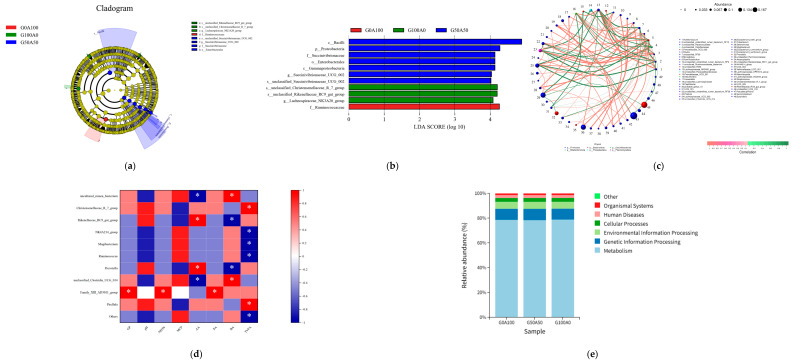
(**a**) LEfSe analysis evolutionary branch diagram (phylum to species; the size of the circle is abundance; yellow is not significant, and the others are classified according to the dominant group). (**b**) LDA value distribution histogram (horizontal axis represents the logarithmic score of LDA analysis, where longer bars represent more significant differences). (**c**) network diagram of each species at genus level (circle size is abundance; red is positively correlated and green is negatively correlated). (**d**) Correlation diagram of silage fermentation parameters and microorganisms. Red was positively correlated, blue was negatively correlated, * showed significant difference (*p* < 0.05). (**e**) Picrust2 function prediction diagram.

Figure 4 shows that the characteristic taxa of the 72 h G0A100 group are *f_Ruminococcaceae*, while the characteristic taxa of the G100A0 group include *s_unclassified_Christensenellaceae_R_7_group*, *s_unclassified_Rikenellaceae_RC9_gut_group*, and *g_Lachnospiraceae_NK3A20_group*. The characteristic taxa of the G50A50 group are *c_Bacilli*, *p_Proteobacteria*, *Succinivibrionaceae*, and *o_Enterobacterales*.

The *unclassified Clostridia UCG_014*, Mogibacterium, and *Rikenellaceae_RC9_gut_group* are densely connected nodes, indicating that these high-abundance groups are the core interaction hubs of the 72 h community, playing a crucial role in the stability of the community structure. Positive correlations far outnumber negative correlations, suggesting a synergistic interaction within the 72-h microbial community. There are both positive and negative correlations between major phyla such as Firmicutes and Bacteroidota, reflecting a complex relationship of synergistic competition among different microbial phyla. In conjunction with previous results, *Rikenellaceae_RC9_gut_group* is a characteristic group of the G100A0 group. Here, it serves as a core interaction hub, indicating that the characteristic groups of the G100A0 group have become key nodes in community interactions, further confirming the significant differentiation of the community in the G100A0 group during the late fermentation phase.

The *Rikenellaceae_RC9_gut_group* and *Prevotella* showed a significant positive correlation with AA. MCP was positively correlated with *unclassified_Clostridia_UCG_014* and negatively correlated with Prevotella, elucidating the bidirectional influence of different bacteria on MCP synthesis. TVFA exhibited a significant positive correlation with *Christensenellaceae_R7_group* and a significant negative correlation with *Ruminococcus*, reflecting the functional differentiation of the microbial community in the later stages of fermentation.

Metabolism is the absolute dominant function among the three groups, with its relative abundance approaching 80%, making it the core function of the microbial community. The secondary functions, such as Genetic Information Processing and Environmental Information Processing, account for less than 20%, serving merely as auxiliary functions. The KEGG functional profiles of the three groups are highly consistent, with no significant differences in the relative abundance of all functional categories between the G0A100, G50A50, and G100A0 groups. This indicates that replacing alfalfa hay with licorice silage only slightly adjusted the composition of microbial groups, while the core functions of the community, especially metabolic functions, did not undergo significant changes. Combined with previous fermentation indicators and nutrient degradation rates, the stability of functional predictions confirms that the core metabolic functions of the microbial community remain stable across different substitution ratios. This stability is key to maintaining the basic fermentation performance of each group, and the functions of the G50A50 group are completely consistent with those of the control group, further supporting its feasibility as a substitute.

## 4. Discussion

GP is an effective predictor of microbial survival and short-chain fatty acid content [37,38]. It is a key indicator reflecting the fermentability of substrates by the rumen microbial community. Generally, the larger the GP, the more thoroughly the substrate is fermented [39]. The G50A50 group showed significant differences in GP at both 3 h and 24 h. Although the difference was not significant at 72 h, the GP value was the highest, indicating a significant difference in IVDMD. This suggests that the G50A50 group promotes the early fermentation initiation in the rumen, making full use of the substrate [40,41,42].

The pH value of the rumen is a crucial factor in regulating the survival of rumen microorganisms. The pH range of the rumen is between 5.5 and 7.5, with the optimal range for microbial fermentation being between 6.6 and 7.2 [43]. In this experiment, the pH of the fermentation liquids in each group remained within the normal range, indicating that the substitution of different proportions of alfalfa hay with 45% semi-dry ensiled licorice stems and leaves did not have any adverse effects on the rumen environment. Microbial protein synthesis and bacterial survival both require conditions of ammonia nitrogen [44]. Research indicates that the optimal range of NH_3_-N concentration is between 3.9 and 290 mg/L. Appropriate levels of NH_3_-N can promote feed intake, the synthesis of microbial protein, and the maintenance of the rumen ecological environment. Conversely, low NH_3_-N concentrations can inhibit the survival of microbial communities [45]. The concentration of NH_3_-N in the rumen indirectly reflects the dynamic relationship between protein degradation in the rumen and microbial protein synthesis [46]. In this study, the NH_3_-N content remained within the normal range, with significant differences observed only at 0 h, indicating that the initial nitrogen release rate of licorice silage is slightly slower than that of alfalfa hay. No significant differences were found among the groups from 3 h to 72 h, suggesting that the microbial efficiency in utilizing nitrogen tends to be consistent across different substitution ratios. In the G100A0 group, the MCP was significantly lower at 0 h and significantly higher at 12 h, indicating that a higher substitution ratio can promote microbial protein synthesis during the mid-fermentation phase, particularly in the G100A0 group. However, while this can enhance MCP, the early pH fluctuations were slightly larger, and the gas production effect was less stable compared to the G50A50 group.

Carbohydrate fermentation primarily produces AA, PA, and BA, which together account for 70–80% of the energy requirements of ruminants [47]. The study of the metabolism of ruminants is crucial, as VFA serve as the primary source of energy and carbon, with one of the main end products being AA [48]. The AA levels in the G50A50 group and the TVFA group were highest at 0 h, indicating a more abundant supply of VFA at the onset of fermentation. After 12 h, the AA content in the G100A0 group was significantly higher than that in the other two groups, suggesting that the fermentation of VFA with 100% substitution was more vigorous in the mid-term. PA plays a crucial role in the synthesis of body fat and lactose and can be converted into glucose through gluconeogenesis in the liver, providing energy to the body; the higher the concentration of PA, the more energy it supplies [49]. In this study, the PA of the control group and the G50A50 group was significantly higher than that of the G100A0 group at 0 h, indicating that the initial VFA release capacity of the G100A0 group was relatively weak. There were no significant differences between the groups in the later fermentation stages, suggesting that the fermentation patterns tended to be consistent.

The in vitro IVDMD of the rumen is a result of microbial decomposition of nutrients, and the degradation rate reflects the production performance of the animal, which is associated with the quality of well-fermented silage [50]. Research indicates that IVNDFD can serve as an important indicator for accurately predicting feed intake, total digestible nutrients, and net energy of forage [49]. In this study, the degradation rates of IVDMD, IVNDFD, and IVADFD in the G50A50 group were close to those of the G0A100 group, with only slight reductions observed at the initial 3 h and 12 h. In contrast, the degradation rate of the G100A0 group consistently remained significantly lower. The findings indicate that when the proportion of licorice stems and leaves in the diet is too high, it inhibits rumen fermentation, which is consistent with the results of this study [51,52]. When G50A50 group occurs, the high-quality protein from alfalfa and the fiber components from licorice create a balance that maintains the substrate utilization efficiency of rumen microbes while avoiding the inhibitory effects on degradation caused by an excessively high proportion of licorice stems and leaves during the substitution.

Principal Component Analysis (PCA) is a dimensionality reduction method that simplifies multiple indicators into a few composite indicators while retaining most of the information from the original indicators, making it more accurate than single evaluations. It is generally accepted that when the eigenvalue of the principal components is greater than 1 and the cumulative variance contribution rate exceeds 85%, the extracted principal components can represent most of the main information of the original variables [53]. This study focuses on three alternative ratio total mixed rations, measuring their fermentation indicators and nutrient degradation characteristics at different times in vitro. PCA was utilized for a comprehensive evaluation, aiming to provide a reference for the comprehensive utilization of Glycyrrhiza stem and leaf. The differences in indicators at different replacement ratios are significant, making it challenging to evaluate their superiority based on a single indicator. Therefore, this study employed PCA to extract principal components from 15 indicators, simplifying them into four composite indicators: F_1_, F_2_, F_3_, and F_4_, with contribution rates of 38.539%, 25.485%, 19.495%, and 6.919%, respectively, resulting in a cumulative contribution rate of 90.437%. Using the contribution rates of these four principal components as weights, a comprehensive evaluation function was constructed, and the comprehensive evaluation values for different substitution ratios were calculated, ranked from highest to lowest as G0A100 > G50A50 > G100A0.

The correlation degree value approaching 1 indicates a higher compatibility of the group with the ‘ideal optimal state’ and a better overall performance. The comprehensive evaluation results of the grey relational degree further validate the feasibility of the G50A50 group as a substitute: the G50A50 group ranks first with a correlation degree of 0.792, outperforming the other two groups and even surpassing the G0A100 group. This result is highly consistent with the previous findings on fermentation parameters and nutrient degradation rates. The G50A50 group demonstrates superior early gas production efficiency (significantly increased gas production at 3 h and 24 h) while maintaining a nutrient degradation level comparable to the control group (no significant differences in IVDMD and IVADFD compared to the G0A100 group). In contrast, the G100A0 group has the lowest correlation degree (0.681), which corresponds to a significant decrease in its nutrient degradation rate and exacerbated functional differentiation of microbial communities in the later stages of fermentation, indicating that G100A0 group disrupts the overall coordination of the rumen fermentation system. In summary, the comprehensive evaluation results of the grey relational degree complement the single analysis of previous indicators, systematically supporting the conclusion that the G50A50 group of licorice stem and leaf silage as a substitute for alfalfa hay represents the optimal ratio balancing overall performance and application value.

Alpha diversity reflects the richness, evenness, and diversity of rumen microbiota. A higher ACE and Chao1 index indicates a greater number of species, while a Simpson index closer to 1 suggests a more uniform species distribution. Additionally, a higher Shannon index indicates greater species diversity [54]. Research has shown that higher pH values correlate with greater biodiversity [55]. In this study, the pH and Shannon index of the G100A0 group after 72 h were significantly higher than those of the other two groups, which is consistent with the findings of this research. However, in conjunction with the previous comprehensive fermentation performance rankings (where the 100% replacement ranked third), we speculate that an increase in species diversity does not equate to superior fermentation functionality; high diversity may accompany redundant species that do not enhance core fermentation efficiency. For most time points, richness, evenness, and diversity showed no significant differences, suggesting that the substitution of alfalfa hay with licorice silage has a minimal impact on the overall structure of the rumen microbial community. Firmicutes are associated with various extracellular enzymes and all types of proteases, lipases, and cellulases, which can degrade macromolecules such as proteins, lipids, carbohydrates, and fibers [56]. Bacteroidota can hydrolyze complex organic matter and then utilize it to synthesize volatile fatty acids, playing a crucial role in maintaining normal gastrointestinal function [57,58,59]. Research indicates that the dominant phyla of rumen bacteria in ruminants are Firmicutes and Bacteroidota, which is consistent with the results of this study [60,61,62]. In this study, when semi-dry ensiled licorice stems and leaves were used as a substitute for alfalfa, an increase in the relative abundance of Bacteroidota was observed. This change corresponds with the elevated production of propionate, as most Bacteroidetes are involved in the conversion of succinate to propionate. The relative abundances of *Prevotella* and *Rikenellaceae_RC9_gut_group*, belonging to the phylum Bacteroidetes, fluctuated significantly before and after fermentation. At 3 h, the control group showed a significantly higher abundance of *Rikenellaceae_RC9_gut_group* compared to the G50A50 group, while at 24 h, the G100A0 group exhibited a significantly higher abundance than the control group. Numerous studies have indicated that these genera are core microbial members of the rumen [63,64]. They play a crucial role in rumen fermentation, and the dynamic changes of this genus reflect the time lag in the activation of carbohydrate-degrading bacteria based on the substitution ratio. In this study, the *Rikenellaceae_RC9_gut_group*, belonging to a high-abundance group, serves as a core interaction hub in the 72 h community, playing a key role in the stability of community structure, which is consistent with the findings of this study. The *Rikenellaceae_RC9_gut_group* plays a crucial role in fiber digestion by secreting a large number of enzymes that degrade cellulose and hemicellulose in the feed, and it is also capable of degrading plant-derived polysaccharides [65,66]. In this study, the IVNDFD of the G0A100 and G50A50 groups was significantly higher than that of the G100A0 group at 12 h. Additionally, the IVADFD of the G0A100 and G50A50 groups was significantly higher than that of the G100A0 group at 12, 24, and 72 h. The reason for this result may be that an increase in abundance does not equate to immediate functional effectiveness. This also indicates that the enrichment of this bacterial genus is a key microbial driver for the enhanced fiber degradation capability of the G50A50 group. The lower IVNDFD and IVADFD in the G100A0 group may be attributed to the potential inhibitory effect of licorice stems and leaves on fiber-degrading bacteria when their proportion in the diet is too high, which can suppress rumen fermentation [67]. We speculate that the inhibitory effects may be due to secondary metabolites such as saponins and tannins present in the licorice stems and leaves [52]. *Prevotella* plays a role in the degradation and utilization of non-cellulosic polysaccharides, proteins, starch, and xylan in plants [68]. The variation in the abundance of *Prevotella* species promotes feed fermentation, and the monosaccharides are the products of their combined enzymatic degradation, which are then directly fermented into volatile fatty acids [69]. In this study, the total volatile fatty acids (TVFA) in the G0A100 and G50A50 groups were significantly higher than those in the G100A0 group. Previous research has indicated that *Prevotella* is a core driving bacterium for the production of volatile fatty acids, and there is a significant positive correlation between *Prevotella* and AA, which aligns with the results of this study [70]. Research indicates that *Rikenellaceae_RC9_gut_group* is positively correlated with pH, while *NK4A214_group* shows a negative correlation with pH, which is consistent with the findings of this study [67,71]. The variation in feed fiber affects the composition of the rumen microbial community, which in turn influences fermentation products and rumen pH. Fiber-degrading bacteria are particularly sensitive to significant changes in rumen pH [72]. When the pH is at 6.8, the cellulose degradation rate of the inoculated rumen culture is the highest [73], which may explain the significantly higher nutrient degradation rates observed in the G0A100 and G50A50 groups. The *Rikenellaceae_RC9_gut_group* has also been reported to be negatively correlated with in vitro rumen fermentation parameters, including BA concentration, while the *Christensenellaceae_R7_group* shows a significant positive correlation with TVFA, which is consistent with the results of this study [74,75]. This indicates that multiple bacterial genera collaboratively maintain the dynamic balance of the in vitro fermentation system. *Ruminococcus* is a key genus of fiber-degrading bacteria that can effectively digest hemicellulose and cellulose [76]. In this study, LEfSe analysis identified the characteristic taxon of the G0A100 group as *f_Ruminococcaceae*, which corresponds with the significantly higher results of IVDFD and IVADFD in the G0A100 group. The proportion of the 3 h G0A100 group was significantly higher than that of the other groups, indicating that alfalfa hay is more likely to activate *Ruminococcus* in the initial stage. The G50A50 group exhibited a significant increase at 24 h, suggesting that the activity of fiber-degrading bacteria in the G50A50 group is enhanced in the mid-stage, which corresponds to the previously observed improvement trend in IVNDFD. The *Lachnospiraceae NK3A20* group plays a crucial role in fiber degradation and the fermentation of plant fibers [77]. Research has also shown that uronic acids account for a significant proportion of legume feed, and the Rumen *Lachnospiraceae isolate NK3A20* produces reduced amounts of H_2_ and CH_4_ when grown on galacturonic acid [78]. In this study, the *Lachnospiraceae_NK3A20_group* was significantly higher in the G50A50 and G100A0 groups at 24 h compared to the G0A100 group. At 72 h, the G100A0 group was significantly higher than the other groups. This indicates that multiple genera are collaboratively involved in the degradation of fibers, which also corroborates the improvement in fiber degradation observed in the G50A50 group at 24 h, suggesting that H_2_ and CH_4_ emissions may also be reduced. *Succinivibrionaceae* are abundant in high-yielding dairy cows, and their presence is positively correlated with milk production and milk fat percentage, while also reducing methane emissions [64,79,80]. Succinivibrionaceae also play an important role in the efficient utilization of nitrogen and environmental protection [81]. In this study, LEfSe analysis identified the microbial biomarker of the G50A50 group as Succinivibrionaceae (recognized at the family, genus, and species levels), indicating that partial replacement of alfalfa hay may reduce gas production and emissions in animals.

KEGG functional prediction indicates that the core functions (metabolism) of the three groups account for nearly 80%, and the functional profiles are highly consistent. This consistency is crucial for maintaining the basic fermentation performance across different substitute groups. The phenomenon of community variation and functional stability essentially reflects the functional redundancy of rumen microorganisms. Although the substitution ratios have altered the composition of some taxa, the core metabolic pathways (such as glycolysis and VFA synthesis) are collectively supported by multiple genera. When the type and quality of feed change, the rumen microbial community can rapidly adjust to maintain the digestion efficiency of nutrients such as fiber, thus leading to no significant changes in community function [82,83]. The functionality of the G50A50 group is essentially consistent with that of the 0% group, further supporting its feasibility as a substitute. At this ratio, a balance between community and functionality has been achieved.

Based on the cost-effectiveness estimation of the G50A50 substitution ratio, taking a typical dairy farm with 100 Holstein cows as an example (with an average daily feed intake of 25 kg per cow, including 4 kg of alfalfa hay), if 50% of the alfalfa hay (i.e., 2 kg per cow per day) is replaced with semi-dry silage made from licorice stems and leaves, the feeding costs can be significantly reduced. According to local market prices, the average daily cost savings per cow is estimated to be between 2.28 and 2.70 RMB (with alfalfa hay priced at 1467–1760 RMB/ton and licorice stems and leaves silage at 327–409 RMB/ton). Annually, the total cost savings for 100 cows can reach 83,000 to 98,000 RMB (based on a full year of 365 days). Additionally, the in vitro fermentation performance and nutritional degradation rate of the G50A50 group are comparable to those of the G0A100 group, indicating economic feasibility for farmers. Further validation through in vivo feeding trials is required.

This study is based on in vitro fermentation experiments; however, it cannot fully simulate the true environment of the rumen, including rumen dynamics, host-microbe interactions, and gastrointestinal physiological mechanisms. Subsequent research should validate the 50% substitution ratio through in vivo feeding trials to assess its impact on biochemical indicators in dairy cows, production performance (milk yield, milk protein, milk fat percentage, etc.), and the long-term health status of the rumen. Additionally, a comprehensive cost benefit analysis should be conducted in conjunction with actual farming scenarios to provide more substantial support for the large-scale application of semi-dry silage from licorice stems and leaves in dairy farming. Furthermore, the fermentation quality of licorice stem and leaf silage may be affected by silage conditions (such as the use of additives and fermentation days); therefore, optimizing the silage preparation process is another important research direction.

## 5. Conclusions

In summary, the optimal substitution ratio of semi-dry ensiled licorice stems and leaves is 50%. Different substitution ratios have varying effects on the microbial community, while the core metabolic functions of the community remain unaffected by the substitution ratio. The total mixed ration that replaces alfalfa hay with 50% semi-dry ensiled licorice stems and leaves can achieve resource substitution for alfalfa hay while ensuring in vitro fermentation performance, nutrient degradation efficiency, and microbial community stability. This provides a feasible solution for the diversification and cost reduction of feed for ruminants.

## Figures and Tables

**Table 1:** Nutritional Value of Semi-Dry Silage of Licorice Stems and Leaves Compared to Alfalfa Hay (DM basis) %.

Items	DM	CP	NDF	ADF	Ca	TP
licorice silage	54.56	14.00	40.82	25.92	0.33	0.18
alfalfa hay	88.7	13.10	45.30	34.10	1.40	0.44

**Table 2:** Composition and nutrient levels of diets (DM basis) %.

Items	Groups		
	G0A100	G50A50	G100A0
Ingredients (%)			
Whole corn silage	8.00	8.00	8.00
wheat straw	12.00	12.00	12.00
oat hay	10.00	10.00	10.00
alfalfa hay	20.00	10.00	0.00
licorice silage	0.00	10.00	20.00
corn	10.00	8.54	3.86
bran	17.50	15.00	17.11
cottonseed cake	17.45	20.77	22.63
beet pulp	1.55	1.29	1.61
CaHPO_4_	1.50	2.40	2.80
NaHCO_3_	1.00	1.00	1.00
Premix ^1^	1.00	1.00	1.00
Total	100.00	100.00	100.00
Nutrient levels ^2^			
CP	16.42	16.63	16.71
EE	2.29	3.00	3.7
NDF	31.44	29.19	27.83
ADF	18.25	18.29	17.5
Ash	7.07	7.31	7.73
Ca	0.92	1.04	1.04
TP	0.61	0.66	0.69
ME	7.24	7.26	7.28

^1^ Each kilogram of the premix contains 370,000 IU of Vitamin A, 3,130,000 IU of Vitamin D, 750 IU of Vitamin E, 110 g of Calcium, 550 mg of Copper, 3400 mg of Zinc, and 1800 mg of Manganese. ^2^ The net energy for lactation is a calculated value, while the other nutritional levels are measured values.

**Table 3:** The effect of different moisture levels of semi-dry ensiled licorice stems and leaves on the gas production during in vitro fermentation (mL).

Items ^1^	Groups			SEM	*p*-Value
	G0A100	G50A50	G100A0		
3 h	14.67 ^b^	19.00 ^a^	16.33 ^ab^	0.75	0.023
6 h	25.00	28.33	26.33	0.67	0.108
9 h	33.00	35.33	33.00	0.70	0.334
12 h	36.33	39.33	36.67	0.71	0.168
24 h	42.33 ^b^	47.33 ^a^	43.00 ^b^	0.97	0.041
36 h	45.67	49.33	44.33	1.18	0.215
48 h	47.33	51.00	49.33	0.83	0.207
72 h	49.00	52.67	51.00	0.87	0.252
A/mL	1.34	6.52	6.14	1.15	0.107
B/mL	53.27	53.46	55.00	1.22	0.856
A + B/mL	54.61	59.97	61.15	1.76	0.304
C	0.29	0.26	0.22	0.02	0.283

^1^ A—rapid gas production phase; B—slow gas production phase; A + B—potential gas production; C—gas production rate constant; different lowercase letters indicate significant differences (*p* < 0.05), while the same lowercase letters and the absence of letters indicate no significant differences (*p* > 0.05), the following table is the same.

**Table 4:** Effects of different moisture levels of semi-dry ensiled licorice stems and leaves on in vitro fermentation pH, NH_3_-N, and MCP.

Items	Groups			SEM	*p*-Value
	G0A100	G50A50	G100A0		
pH					
0 h	7.25	7.22	7.23	0.01	0.724
3 h	7.11 ^a^	7.06 ^b^	7.12 ^a^	0.01	0.022
12 h	7.02 ^b^	7.06 ^a^	7.01 ^b^	0.01	0.033
24 h	7.01	6.99	7.00	0.01	0.643
72 h	6.88 ^b^	6.88 ^b^	6.90 ^a^	0.00	0.000
NH_3_-N/mg·L^−1^					
0 h	116.50 ^a^	101.87 ^b^	111.23 ^a^	2.45	0.014
3 h	119.17	118.34	118.34	1.76	0.981
12 h	120.68	122.52	120.18	0.95	0.635
24 h	137.31	139.07	141.66	0.86	0.100
72 h	184.04	196.32	184.37	3.22	0.223
MCP/mg·L^−1^					
0 h	18.06 ^a^	17.67 ^a^	12.72 ^b^	0.91	0.001
3 h	19.25	19.74	23.70	1.13	0.227
12 h	23.21 ^b^	26.38 ^ab^	28.95 ^a^	0.96	0.017
24 h	27.17	32.31	32.41	1.16	0.088
72 h	23.11	23.11	21.03	0.87	0.598

Different lowercase letters indicate significant differences (*p* < 0.05), while the same lowercase letters and the absence of letters indicate no significant differences (*p* > 0.05).

**Table 5:** The Effect of Different Moisture Levels of Semi-Dry Silage from Licorice Stems and Leaves on In Vitro Fermentation VFA.

Items ^1^	Groups			SEM	*p*-Value
	G0A100	G50A50	G100A0		
AA/mmol·L^−1^					
0 h	12.28 ^b^	14.31 ^a^	10.16 ^c^	0.66	0.005
3 h	14.58	14.53	16.00	0.62	0.620
12 h	18.92 ^b^	18.73 ^b^	23.94 ^a^	1.00	0.019
24 h	27.34	27.74	29.67	0.62	0.284
72 h	31.62	31.50	32.16	1.10	0.975
PA/mmol·L^−1^					
0 h	6.73 ^a^	7.06 ^a^	5.67 ^b^	0.23	0.004
3 h	7.73	8.54	8.28	0.33	0.666
12 h	9.48	9.46	10.40	0.31	0.434
24 h	12.34	12.03	12.45	0.27	0.842
72 h	14.06	14.26	14.14	0.37	0.982
BA/mmol·L^−1^					
0 h	0.77 ^a^	0.90 ^a^	0.48 ^b^	0.07	0.013
3 h	1.04	1.25	1.08	0.09	0.707
12 h	1.87	1.80	1.97	0.10	0.826
24 h	3.19	3.04	3.00	0.11	0.802
72 h	3.69	3.78	3.55	0.16	0.875
TVFA/mmol·L^−1^					
0 h	19.79 ^a^	22.26 ^a^	16.31 ^b^	0.94	0.004
3 h	23.36	24.32	25.36	0.98	0.759
12 h	30.27	29.98	36.31	1.32	0.060
24 h	42.87	42.80	45.13	0.76	0.424
72 h	49.37	49.54	49.85	1.53	0.994

^1^ AA—Acetic acid; PA—Propionic acid; BA—Butyric acid; TVFA—Total volatile fatty acids. Different lowercase letters indicate significant differences (*p* < 0.05), while the same lowercase letters and the absence of letters indicate no significant differences (*p* > 0.05).

**Table 6:** Effect of in vitro fermentation of licorice stem and leaf semi-dry silage instead of alfalfa hay on nutrient degradation rate.

Items ^1^	Groups			SEM	*p*-Value
	G0A100	G50A50	G100A0		
IVDMD%					
3 h	28.96 ^a^	24.87 ^b^	20.21 ^c^	1.27	0.000
12 h	39.63 ^a^	34.57 ^b^	30.33 ^c^	1.36	0.000
24 h	47.78 ^a^	46.50 ^a^	40.14 ^b^	1.34	0.012
72 h	53.33 ^a^	52.90 ^a^	46.22 ^b^	1.24	0.003
IVNDFD%					
3 h	11.35	10.25	9.40	0.45	0.224
12 h	20.30 ^a^	19.22 ^a^	17.62 ^b^	0.44	0.012
24 h	29.71	28.47	27.51	0.42	0.080
72 h	42.52	41.90	38.17	0.91	0.086
IVADFD%					
3 h	10.40	10.20	9.39	0.28	0.336
12 h	13.84 ^a^	12.40 ^a^	10.42 ^b^	0.56	0.012
24 h	24.12 ^a^	23.14 ^a^	19.39 ^b^	0.76	0.001
72 h	35.81 ^a^	35.01 ^a^	33.27 ^b^	0.45	0.029

^1^ IVDMD—In vitro dry matter degradation rate; IVNDFD—In vitro neutral detergent fiber degradation rate; IVADFD—In vitro acid detergent fiber degradation rate. Different lowercase letters indicate significant differences (*p* < 0.05), while the same lowercase letters and the absence of letters indicate no significant differences (*p* > 0.05).

**Table 7:** The characteristic values of the principal components and their variance contributions.

	Initial Eigenvalue			Extracted Sum of Squares of Load			Sum of Squares of Rotational Loads		
Principak Component Number	Eigenvalue	Percentage of Variance (%)	Cumulative (%)	Eigenvalue	Percentage of Variance (%)	Cumulative (%)	Eigenvalue	Percentage of Variance (%)	Cumulative (%)
1	5.781	38.539	38.539	5.781	38.539	38.539	4.219	28.129	28.129
2	3.823	25.485	64.023	3.823	25.485	64.023	4.204	28.029	56.158
3	2.924	19.495	83.518	2.924	19.495	83.518	3.83	25.532	81.69
4	1.038	6.919	90.437	1.038	6.919	90.437	1.312	8.747	90.437
5	0.882	5.881	96.318						
6	0.441	2.938	99.255						
7	0.087	0.579	99.834						
8	0.025	0.166	100						
9	5.568 × 10^−16^	3.712 × 10^−15^	100						
10	2.916 × 10^−16^	1.944 × 10^−15^	100						
11	8.372 × 10^−17^	5.581 × 10^−16^	100						
12	−1.52 × 10^−16^	−1.02 × 10^−15^	100						
13	−2.87 × 10^−16^	−1.91 × 10^−15^	100						
14	−3.78 × 10^−16^	−2.52 × 10^−15^	100						
15	−7.83 × 10^−16^	−5.22 × 10^−15^	100						

**Table 8:** The principal component loading matrix and characteristic vectors.

	First Principal Components		Second Principal Components		Third Principal Components		Fourth Principal Components	
Index	Loads	Eigenvectors	Loads	Eigenvectors	Loads	Eigenvectors	Loads	Eigenvectors
E_1_	0.196	0.082	0.087	0.044	−0.557	−0.326	0.656	0.644
E_2_	0.629	0.262	−0.374	−0.191	−0.414	−0.242	−0.514	−0.505
E_3_	−0.572	−0.238	0.329	0.168	−0.103	−0.060	−0.058	−0.057
E_4_	−0.684	−0.284	−0.179	−0.092	0.021	0.012	0.450	0.442
E_5_	0.853	0.355	0.473	0.242	0.152	0.089	0.086	0.084
E_6_	0.426	0.177	0.782	0.400	−0.370	−0.216	0.027	0.027
E_7_	0.425	0.177	0.893	0.457	−0.114	−0.067	0.003	0.003
E_8_	0.762	0.317	0.624	0.319	0.007	0.004	0.069	0.068
E_9_	0.738	0.307	−0.160	−0.082	0.629	0.368	0.112	0.110
E_10_	0.724	0.301	−0.295	−0.151	0.603	0.353	0.111	0.109
E_11_	−0.764	−0.318	0.047	0.024	−0.616	−0.360	−0.080	−0.079
E_12_	−0.212	−0.088	0.915	0.468	−0.239	−0.140	−0.149	−0.146
E_13_	−0.553	−0.230	0.477	0.244	0.665	0.389	0.125	0.123
E_14_	−0.465	−0.193	0.482	0.247	0.656	0.384	−0.229	−0.225
E_15_	−0.812	−0.338	0.316	0.162	0.436	0.255	−0.048	−0.047

**Table 9:** Comprehensive score.

Groups	F_1_	F_2_	F_3_	F_4_	F ^1^	Rank
G0A100	0.188	−0.054	0.640	−0.431	0.170	1
G50A50	−0.235	0.191	0.642	0.638	0.141	2
G100A0	0.047	−0.137	−1.282	−0.207	−0.311	3

^1^ F represents the comprehensive evaluation score calculated by PCA, with the formula: F = (0.38539F_1_ + 0.25485F_2_ + 0.19495F_3_ + 0.06919F_4_)/0.90437.

**Table 10:** Comprehensive analysis of grey correlation degree.

Groups	Correlation Degree	Rank
G0A100	0.756	2
G50A50	0.792	1
G100A0	0.681	3

**Table 11:** Analysis of α-diversity of rumen microorganisms during in vitro fermentation of semi-dry silage of licorice stems and leaves at different moisture levels.

Items	Groups			SEM	*p*-Value
	G0A100	G50A50	G100A0		
ACE					
0 h	5747.22	5101.43	5658.18	621.90	0.913
3 h	5468.40	5138.14	4889.50	522.15	0.915
12 h	5723.82	5971.65	5810.35	496.82	0.982
24 h	3142.68	3827.29	6057.11	564.29	0.077
72 h	3156.32	3839.85	5679.22	471.59	0.066
Chao1					
0 h	4194.06	3949.83	4177.20	400.19	0.967
3 h	4313.21	3941.73	3799.17	330.64	0.829
12 h	4392.02	4401.41	4412.49	315.91	1.000
24 h	2817.05	3126.38	4780.41	384.46	0.069
72 h	2926.14	3173.82	4602.20	324.55	0.063
Simpson					
0 h	0.98	0.99	0.99	0.00	0.090
3 h	0.98	0.99	0.99	0.00	0.117
12 h	0.99	0.99	0.99	0.00	0.734
24 h	0.97	0.98	0.99	0.01	0.559
72 h	0.99	0.99	1.00	0.00	0.172
Shannon					
0 h	8.78	9.28	9.09	0.20	0.609
3 h	9.17	9.43	8.96	0.20	0.664
12 h	9.50	9.22	9.27	0.18	0.820
24 h	8.47	8.63	9.73	0.32	0.230
72 h	9.10 ^b^	8.91 ^b^	10.00 ^a^	0.20	0.047

Different lowercase letters indicate significant differences (*p* < 0.05), while the same lowercase letters and the absence of letters indicate no significant differences (*p* > 0.05).

**Table 12:** The horizontal distribution of rumen microbial phylum in in vitro fermentation of licorice stem and leaf semi-dry silage instead of alfalfa hay.

Items	Groups			*p*-Value
	G0A100	G50A50	G100A0	
Firmicutes				
0 h	60.84	66.50	63.54	0.904
3 h	54.24	67.24	61.06	0.583
12 h	54.96	53.48	57.06	0.951
24 h	78.33	73.16	55.48	0.083
72 h	80.77	76.92	64.43	0.113
Bacteroidota				
0 h	28.92	29.23	37.45	0.51
3 h	9.20 ^b^	43.54 ^a^	31.35 ^a^	0.019
12 h	26.17	34.26	42.74	0.416
24 h	25.55	21.10	30.46	0.839
72 h	5.01 ^b^	22.56 ^a^	30.13 ^a^	0.013
Planctomycetota				
0 h	7.14	2.76	7.28	0.376
3 h	4.29	3.85	4.27	0.978
12 h	2.38	3.33	2.95	0.863
24 h	0.88	3.26	3.87	0.295
72 h	0.82	1.55	3.54	0.06
Proteobacteria				
0 h	1.92	2.4	1.68	0.88
3 h	0.72	1.6	3.17	0.307
12 h	0.96	2.00	3.10	0.28
24 h	3.22	2.65	0.62	0.192
72 h	2.72 ^ab^	4.12 ^a^	0.68 ^b^	0.049
Cyanobacteria				
0 h	0.18	0.34	0.81	0.456
3 h	0.10	0.57	0.29	0.171
12 h	0.09 ^b^	1.19 ^a^	0.14 ^b^	0.024
24 h	0.64	0.18	0.15	0.057
72 h	1.44	1.35	0.41	0.144
Desulfobacterota				
0 h	0.15	0.35	0.16	0.168
3 h	0.29	0.38	0.15	0.284
12 h	0.38	0.26	0.35	0.761
24 h	0.27 ^b^	0.07 ^b^	0.87 ^a^	0.003
72 h	0.17 ^b^	0.22 ^b^	0.74 ^a^	0.001
Spirochaetota				
0 h	0.08	0.2	0.06	0.082
3 h	0.09 ^b^	0.47 ^a^	0.13 ^b^	0.031
12 h	0.54	0.54	0.27	0.586
24 h	0.5	0.08	0.34	0.081
72 h	0.1	0.25	0.3	0.098

Different lowercase letters indicate significant differences (*p* < 0.05), while the same lowercase letters and the absence of letters indicate no significant differences (*p* > 0.05).

**Table 13:** The horizontal distribution of rumen microorganisms in vitro fermentation of licorice stem and leaf semi-dry silage instead of alfalfa hay.

Items	Groups			*p*-Value
	G0A100	G50A50	G100A0	
uncultured_rumen_bacterium				
0 h	14.58	13.75	11.35	0.946
3 h	5.44	25.19	16.83	0.130
12 h	6.54	15.81	14.21	0.510
24 h	21.86	19.78	7.29	0.230
72 h	20.67	26.06	11.41	0.312
Christensenellaceae_R_7_group				
0 h	14.48	13.72	17.15	0.801
3 h	17.87	11.42	10.52	0.205
12 h	16.31	13.58	14.64	0.896
24 h	11.26	11.55	21.13	0.134
72 h	10.87	11.09	22.33	0.082
Rikenellaceae_RC9_gut_group				
0 h	15.61	9.00	14.14	0.444
3 h	22.38 ^a^	8.77 ^b^	12.20 ^ab^	0.046
12 h	16.22	13.76	11.80	0.614
24 h	6.26 ^b^	10.01 ^ab^	19.32 ^a^	0.046
72 h	8.22	7.63	16.48	0.232
Prevotella				
0 h	9.60	13.25	7.73	0.559
3 h	13.10	4.76	13.00	0.163
12 h	20.06	16.82	17.87	0.865
24 h	6.32	7.33	12.74	0.288
72 h	3.33	2.01	4.62	0.316
Ruminococcus				
0 h	3.35	2.05	4.76	0.098
3 h	4.23 ^a^	2.22 ^b^	2.55 ^b^	0.002
12 h	2.22	2.18	2.10	0.938
24 h	1.41 ^c^	3.49 ^a^	1.87 ^ab^	0.049
72 h	5.13	3.40	3.23	0.097
Lachnospiraceae_NK3 A20_group				
0 h	2.23	2.66	1.78	0.789
3 h	1.59	2.49	1.31	0.522
12 h	2.01	1.03	2.81	0.119
24 h	0.46 ^c^	1.57 ^ab^	3.15 ^a^	0.034
72 h	0.46 ^b^	0.52 ^b^	2.55 ^a^	0.004

Different lowercase letters indicate significant differences (*p* < 0.05), while the same lowercase letters and the absence of letters indicate no significant differences (*p* > 0.05).

## Data Availability

The data presented in this study are openly available in the NCBI repository at https://www.ncbi.nlm.nih.gov/, or PRJNA1392334.
